# A multi-site, multi-participant magnetoencephalography resting-state dataset to study dementia: The BioFIND dataset

**DOI:** 10.1016/j.neuroimage.2022.119344

**Published:** 2022-05-31

**Authors:** Delshad Vaghari, Ricardo Bruna, Laura E. Hughes, David Nesbitt, Roni Tibon, James B. Rowe, Fernando Maestu, Richard N. Henson

**Affiliations:** 1 MRC Cognition & Brain Sciences Unit, University of Cambridge, UK; 2 Department of Electrical & Computer Engineering, Tarbiat Modares University; 3 Department of Experimental Psychology, Complutense University of Madrid; 4 Laboratory of Cognitive and Computational Neuroscience (UCM-UPM), Center for Biomedical Technology; 5 Cambridge University Hospitals NHS Trust and Department of Clinical Neurosciences, University of Cambridge, UK; 6 Department of Psychiatry, University of Cambridge, UK

## Abstract

Early detection of Alzheimer’s Disease (AD) is vital to reduce the burden of dementia and for developing effective treatments. Neuroimaging can detect early brain changes, such as hippocampal atrophy in Mild Cognitive Impairment (MCI), a prodromal state of AD. However, selecting the most informative imaging features by machine-learning requires many cases. While large publically-available datasets of people with dementia or prodromal disease exist for Magnetic Resonance Imaging (MRI), comparable datasets are missing for Magnetoencephalography (MEG). MEG offers advantages in its millisecond resolution, revealing physiological changes in brain oscillations or connectivity before structural changes are evident with MRI. We introduce a MEG dataset with 324 individuals: patients with MCI and healthy controls. Their brain activity was recorded while resting with eyes closed, using a 306-channel MEG scanner at one of two sites (Madrid or Cambridge), enabling tests of generalization across sites. A T1-weighted MRI is provided to assist source localisation. The MEG and MRI data are formatted according to international BIDS standards and analysed freely on the DPUK platform (https://portal.dementiasplatform.uk/Apply). Here, we describe this dataset in detail, report some example (benchmark) analyses, and consider its limitations and future directions.

## Introduction

Alzheimer’s disease (AD) is an age-related neurodegenerative disorder that is characterised by progressive dementia, from mild memory impairment to global cognitive dysfunction and eventually death (Masters et al., 2015). According to the World Alzheimer report in 2019, there are 50 million people in the world with dementia, which is likely to rise to 152 million people by 2050 (Wimo et al., 2003). This prevalence accentuates the need for reliable biomarkers that are sensitive to the early stages of the disease. Although there is currently no cure for AD, early detection may enable more effective management and the ability to prevent or delay dementia. Biomarkers that are accurate, safe, and sensitive to the specific brain changes in dementia are required to accelerate and increase power for early phase clinical trials.

Here we consider the challenge of Mild Cognitive Impairment (MCI), which is commonly a prodromal state of AD, with a high probability of progression to dementia (Bruscoli and Lovestone, 2004; Kelley and Petersen, 2007). MCI is defined by cognitive symptoms and performance on cognitive tests, with or without specific biomarker evidence of underlying AD pathology (Winblad et al., 2004). Although a prodromal disorder, patients may have subtle brain changes that are identifiable with neuroimaging. Neuroimaging offers a range of potential biomarkers of structural, metabolic and functional changes in the brain related to MCI and AD (Cabeza et al., 2018; Tartaglia et al., 2011; Woo et al., 2017). The dominant form of neuroimaging is Magnetic Resonance Imaging (MRI), which is most often used clinically to measure brain structure, particularly the volume of grey-matter in brain regions susceptible to AD, such as in the medial temporal lobes (Frisoni et al., 2010). However, atrophy is a late pathological stage of neurodegenerative disease, occurring potentially many years after molecular and physiological changes (Dubois et al., 2016; Han et al., 2012; Jack et al., 2017, 2013). While functional change can be quantified by functional-MRI, the latter is subject to neurovascular confounds, motion artefacts and low-reliability (Tsvetanov et al., 2021).

Magnetoencephalography (MEG) has been proposed as a valuable alternative tool for functional biomarkers of early-stage AD. MEG has a better temporal resolution to measure brain function, is reliable across sessions (Colclough et al., 2016; Garcés et al., 2016; Martín-Buro et al., 2016) and is not confounded by neurovascular variance. While Electroencephalography (EEG) can also measure neural activity directly like MEG, it does not offer the same spatial resolution, or potential to measure changes in the brain’s functional connectome (see (López-Sanz et al., 2017; Maestú et al., 2019), for a more detailed discussion of the potential advantages of MEG for detecting AD).

MEG offers a large set of potential data features that might differentiate individuals with MCI from healthy controls. These features may be limited to specific frequencies of oscillatory activity, specific brain regions, or the functional connectivity between brain regions. The spatiotemporal complexity of MEG is well suited for machine/deep learning techniques to identify those features that enable the classification of MCI (Maestú et al., 2015). However, these techniques typically need a large number of cases to train and test the classifiers. While large datasets of MRI scans of MCI cases have been made available to the community (e.g., (Jack et al., 2008; Mueller et al., 2005), comparable datasets of MEG are required.

In the recent “BioFIND” project, funded by the European Union’s Joint Programming For Neurodegenerative Research initiative (Hughes et al., 2019), we combined 168 MEG datasets from a number of ongoing dementia projects at the University of Cambridge, England, and the Centre for Biomedical Technology in Madrid, Spain. Since then, we have added further data, nearly doubling the total to 324 participants, approximately half of whom had MCI (according to NIA-AA criteria, (Albert et al., 2011), while the rest were healthy controls. Participants contributed 2-13 minutes of resting-state MEG, plus a T1-weighted structural MRI scan in most cases. The MRI can be used to help localise the cortical sources of the MEG data, and also to compare classification based on MEG with that based on the more commonly used structural MRI.

Here we describe this extended BioFIND dataset, in the hope that it will allow others to investigate aspects of brain function that differ in MCI patients versus controls, and hopefully identify potential biomarkers for early AD. While some of the data were reported in our previous paper (Hughes et al., 2019), those data were not made available, nor were they in a shareable format. Here we have converted the data to the international BIDS format (Gorgolewski et al., 2016; Niso et al., 2018), which includes meta-data on a number of other relevant factors, and describe how the data can be analysed on the DPUK platform (Bauermeister et al., 2020) (https://portal.dementiasplatform.uk/Apply). We report an example preprocessing (feature extraction) and classification approach, to confirm basic data quality, but emphasize that this is not supposed to be an optimised approach and that the main aim of this paper is to describe the data in sufficient detail that others can test their own preprocessing and classification approaches.

## Methods

### Participants

The 324 participants consist of 158 people with clinically diagnosed MCI and 166 controls, recorded at one of two sites: 1) the MRC Cognition & Brain Sciences Unit (CBU) at the University of Cambridge, and 2) the Laboratory of Cognitive and Computational Neuroscience at the Centre for Biomedical Technology (CTB), Madrid. The participants were pooled over a number of different projects, each approved by local Ethics Committees and following the 1991 Declaration of Helsinki. Participants consented to the collection and sharing of de-identified data for research purposes.

The 68 MCI patients scanned at Cambridge were recruited from specialist memory clinics at Cambridge University Hospitals NHS Trust. Individuals were diagnosed with MCI after referral for symptoms, mainly memory problems (i.e., they were not derived by screening of cognitively asymptomatic people). The diagnosis was made in a regional memory clinic, including ACE/ACER and MMSE tests as standard, with a significant deficit in memory domain tests. PET and fluidic Biomarkers were not used as standard, although all had structural brain imaging (usually MRI in the clinic unless contraindicated, when a CT was occasionally used) and clinical follow-up in support of the diagnosis. By definition of MCI, sufferers had functional independence at the time of diagnosis. sMRI was used to exclude other pathologies and to identify features consistent with MCI/AD pathology (e.g. MTL atrophy without mass lesion, high vascular burden). Patients had no obvious major psychiatric disorder.

The 91 controls from Cambridge were selected from the population-derived CamCAN cohort of healthy people from the same geographic region (www.cam-can.org), chosen to have similar age and sex distribution. The CBU controls are screened to be healthy, i.e., have MMSE (and indeed ACE-R) scores above conventional cut-offs, as well as other screening described in CamCAN paper (Shafto et al., 2014).

The 90 patients and the 75 controls from Madrid were recruited from the Neurology and Geriatric Departments of the University Hospital San Carlos. The MCI diagnosis of the patients was determined with intermediate probability according to the National Institute on Aging–Alzheimer Association criteria (Albert et al., 2011), i.e., given by a clinician based on clinical and cognitive tests, self- and informant-report, and in the absence of full dementia or obvious other causes. For some patients, there was additional biomarker evidence of atrophy from MRI or long term follow up and genotyping for the *APOE* ε4 allele. The diagnosis required an objective impairment in the memory domain and/or other cognitive functions, but a subjective “memory complaint” was not required, and (as a requirement for the diagnosis criteria), they were able to still perform their daily living activities. MRI (T1, T2 and/or FLAIR) was used to rule out a vascular disorder, and any other type of neurological disease (i.e., tumour, stroke, infection) that could better explain the cognitive symptoms.

The CTB controls had a full neuropsychological assessment to confirm normal cognition, and the same type of MRI assessment as that done in the MCI group, i.e., a radiologist reported MRIs as normal. All participants were free of any significant neurological or psychiatric disorder, including vascular damage (Hachinski score equal or less than 4, plus observation of T2-weighted MRI) and depression (geriatric depression score equal or less than 5), or any medication with known effects on MEG activity.

Note that the MRI (and MEG) data provided here were research scans following diagnosis, and were not used to inform the diagnosis, though other similar (T1-weighted) clinical MRIs may have been used by the diagnosing clinician. For a subset of MCI patients, we indicate whether or not they subsequently progressed to dementia (“probable AD”) over subsequent years, according to their managing clinician.

The distributions of participant sex, age, education, and score on a cognitive test for dementia - the Mini-Mental State Examination (MMSE) - are shown in Table 1. As expected (since part of the diagnosis), the MCI group scored lower on the MMSE, with most below the common clinical threshold of 27 (O’Bryant et al., 2008). While MMSE may lack sensitivity to MCI, its widespread use, approval as a clinical trial outcome and multiple language versions make it a suitable as a screening tool. T-tests confirmed that the MCI group was also slightly older and less well educated on average, though there was considerable variance across patients and appreciable overlap between them and the controls, enabling subgroup matching where relevant to future analyses.

Note that comparable resting-state MEG data (and T1-weighted MRIs) acquired at the CBU site are also available for approximately 600 healthy participants (aged 18-88 years) via the CamCAN website: https://camcan-archive.mrc-cbu.cam.ac.uk/dataaccess/. These data could be used with machine learning to characterise healthy MEG data or predict age, which could then be tested on the present patient data.

### Resting-state Protocol

The MEG data were recorded while participants were asked to keep their eyes closed, instructed to think of nothing specific, but not fall asleep. The duration of these recordings varied from 2 to 13 minutes (Table 1). A Wilcoxon rank-sum test showed that the duration of the median MEG recording was longer in controls. It is therefore recommended that data are trimmed so that the same duration is used for all participants (since levels of drowsiness might increase for longer recordings).

The precise date and time of recording were scrambled in the FIFF file using the “mne_anonymize” function (https://mne.tools/dev/generated/commands.html#mne-anonymize) of the MNE software (Gramfort et al., 2013), to reduce the risk of participant identification. Nonetheless, in case they are relevant to the MEG data quality, the time of day and the year of the recording are provided in the participants.tsv file. As can be seen in Table 1, the time of day (Recoding Hour) did not differ between patients and controls. The data for patients were recorded approximately a year earlier on average than those for controls, though again there was large overlap in the Recoding Year (Table 1). While there is no obvious reason why data quality should change over years, we also provide empty-room data for each year and site, to enable estimation of changes in ambient noise levels.

Some of the participants performed other tasks in the MEG scanner prior to the resting-state phase. The median and range of the duration (in minutes) of such tasks is shown in Table 1, and did not differ significantly between patients and controls. However, performing such tasks could affect the resting-state data (e.g, make participants more tired), so can be used as a confounding covariate. The nature of the tasks varied. For CBU data, sub-Sub0127 to sub-Sub0155 task performed a semantic judgment task on auditory sentences followed by a visual word (as described in (Olichney et al., 2008), while sub-Sub0156 to sub-Sub0168 performed an auditory MisMatch Negativity (MMN) task (as described in (Hughes and Rowe, 2013). For CTB data, if any participant performed a task, it was a delayed-match-to-sample task (as described in (Serrano et al., 2020). For CBU participants for whom tasks were done before the resting-state, the scanner was stopped in between, so the resting-state was a new FIF file. For some CTB participants for whom tasks were done before the resting-state, the scanner was not stopped, so a single FIF file was saved, but a new FIF file for the resting-state section was subsequently extracted using the MNE “trim” function.

### M/EEG data acquisition

MEG recordings were collected continuously at 1 kHz sample rate using an Elekta Neuromag Vectorview 306 MEG system (Helsinki, FI) at both CBU and CTB sites. The MEG data were acquired while participants were seated inside a magnetically shielded room (MSR). The CBU MSR is made by Imedco and uses single layer mu-metal plates, while the Madrid MSR is made by Vaccumschmelze and has two layers. For the CBU, the average MSR noise level during tuning was 2.3 fT/sqrt(Hz); for the CTB, it was 2.8 fT/sqrt(Hz) until 2016, and 2.6 fT/sqrt(Hz) after 2016. The VectorView system includes two orthogonal planar gradiometers and one magnetometer at each of 102 locations around the head. For many but not all participants, bipolar electrodes were used to record the electro-oculogram (EOG), for vertical and/or horizontal eye movements, as well as the electro-cardiogram (“ECG”). When present, these correspond to EEG channels EEG061 (HEOG), EEG062 (VEOG) and EEG063 (ECG) (see ‘Data records’ section). For a smaller subset of CBU participants, an additional 70 channels of nose-referenced, unipolar EEG were recorded, but we do not analyse these data here.

**Figure 1:**
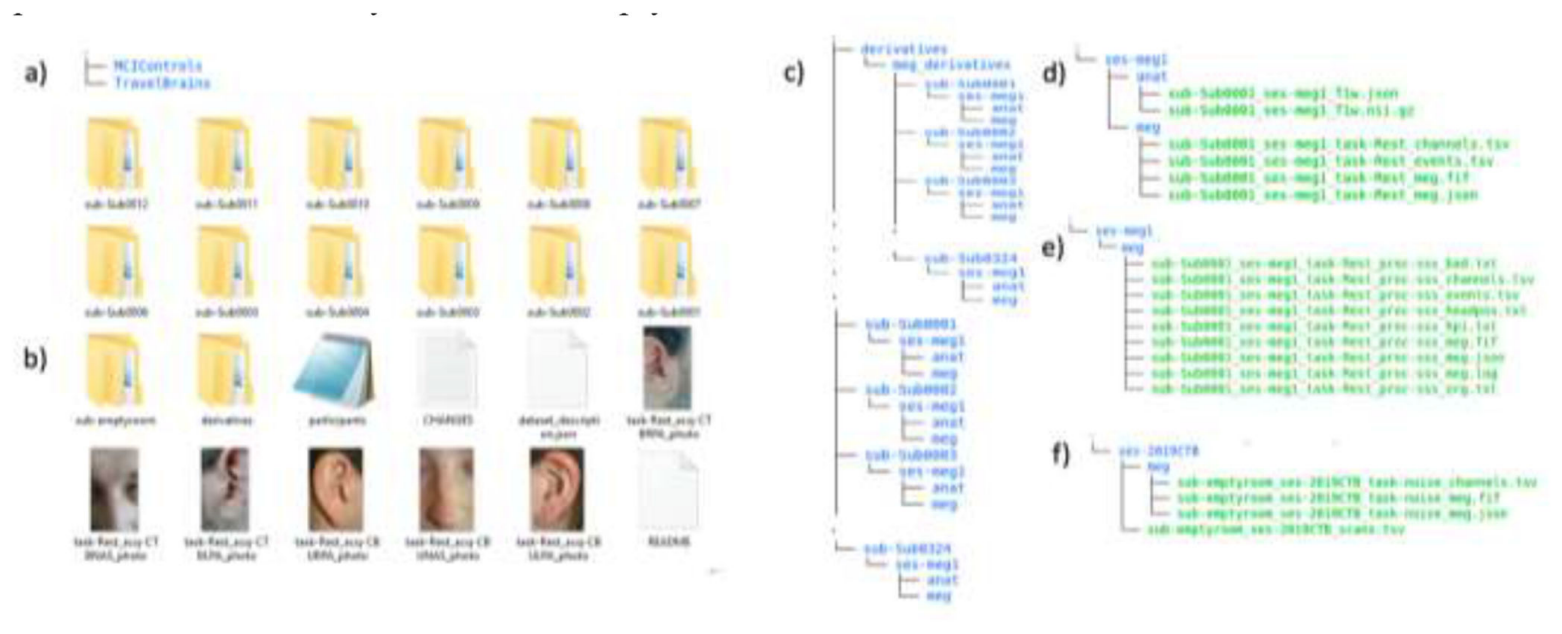
a) Content of Top-level directory b) Organization of MCIControls directory; c) Content of participant-specific directories; d) Content of MRI and MEG data directories for each participant; e) Content of maxfiltered-MEG data directories; f) Content of sub-emptyroom directory

To monitor head position throughout the scan, head position indicator (HPI) coils were attached to the scalp and detected by the MEG machine (energized at frequencies above 150 Hz in CTB and above 300Hz in CBU). Prior to the scan, a Fastrak digitizer (Polhemus Inc., Colchester, VA, USA) was used to record locations of the HPI coils, in addition to three anatomical fiducials, for the Nasion, Left and Right Peri-Auricular points (LPA and RPA respectively), plus approximately 100 points across the scalp (to help coregistration with the MRI). For CBU data, the LPA and RPA refer to pre-auricular points; for CTB data, the LPA and RPA refer to a point anterior to the tragus (photographs of these points are provided in the top-level BIDS directory, as shown in Figure 1b).

### MEG maxfiltering

In addition to the raw data, we also provide versions that have been de-noised using Signal Space Separation (SSS) (Taulu and Kajola, 2005) as implemented in MaxFilter version 2.2.12 (https://imaging.mrc-cbu.cam.ac.uk/meg/Maxfilter_V2.2). The full parameters used by MaxFilter are present in the “log” files described in the “Data Records” section below, but are summarised here. First, a sphere was fit to the digitised head points, excluding points on the nose, and the coordinates of the centre that sphere were passed to MaxFilter. MaxFilter was then called twice: first, just to detect bad channels in each data buffer (using MaxFilter’s “autobad” option), and to estimate head position every second (using MaxFilter’s “headpos” option). In the second call (which produced the log files below), MaxFilter was passed the list of channels that were bad in more than 5% of buffers (for a given participant) and SSS was performed to remove noise from the data (briefly, any spatial modes that arise from magnetic sources outside a sphere that encompassed the sensor array). MaxFilter also recreated data in bad channels on the basis of the remaining channels (using spherical basis functions). Note that MaxFilter’s “temporal” SSS option was not used for further de-noising, given uncertainty about the best temporal parameters; nor was MaxFilter’s “mvcomp” option to correct for head motion, because it failed on some participants; nor was MaxFilter’s “trans” option to align to a common space, given that this is imperfect and more accurate forward models can be created from the MRIs (see later). For SSS, MaxFilter needs site-specific calibration and cross-talk files, which we provide in the “meg_derivatives” sub-directory (see below). All other MaxFilter parameters were kept as their default, as described in the manual available on https://imaging.mrc-cbu.cam.ac.uk/meg/Maxfilter_V2.2 and output to the log files below. The MATLAB script used for maxfiltering is also provided in the accompanying GitHub directory (“maxfilter_BIDS.m” in https://github.com/delshadv/BioFIND-data-paper).

The mean and standard deviation of the head translation estimated by the first call of MaxFilter are shown in Table 1. Neither differed significantly between groups, but these measures of movement could be used as additional covariates when analysing the MEG data.

### MRI data acquisition

T1-weighted MRIs for participants tested at the CBU were acquired on either a Siemens 3T TIM TRIO or Prisma using a magnetization-prepared rapid gradient echo (MP-RAGE) pulse sequence. The T1-weighted MRI for participants tested at the CTB were acquired on a General Electric 1.5 Tesla MRI using a high-resolution antenna with a homogenization PURE filter.

### Data Records

The data are available for analysis on the DPUK’s analysis platform, access to which can be obtained via https://portal.dementiasplatform.uk/Apply (the data are also summarised on the DPUK cohort website, https://doi.org/10.48532/007000). This platform provides virtual desktops to enable analysis on its servers. The reason for sharing via the DPUK servers is to ensure that the data cannot leave those servers, preventing use by others who have not agreed to the DPUK’s data usage agreement, and thereby respecting the consent given by participants. If use of the data is requested that is not possible on the DPUK servers, researchers can contact the corresponding author in order to create and sign a separate “data transfer agreement” with conditions on data transfer and usage.

There is an application form to apply for DPUK access, which once submitted, enables analysis through the above portal. Within a few days after submission, an instruction email with the necessary credentials will be sent to users by DPUK. The user then needs to follow simple instructions and log in using https://portal.dpuk.uksep.ac.uk/. Both Windows and Linux operating systems are provided. The paths to data on Windows and Linux are S:\BioFind - BioFIND\BioFIND\* and /biofind/data/* respectively (see code availability section below). Both operating systems also have MATLAB and python available, for example, and users can request upload of further software, subject to any licencing. Other data can be uploaded to the server using the file in/out request mechanism on https://portal.dpuk.ukserp.ac.uk.

The data are represented in the Brain Imaging Data Structure (BIDS) format (version 1.4.1; http://bids.neuroimaging.io), which is an international, community effort (Gorgolewski et al., 2016; Niso et al., 2018). DPUK required the files to be originally uploaded in XNAT format (https://www.xnat.org/). However, we subsequently converted them to the BIDS format using the script called Xnat2Bids.m, which can be found in the GitHub repository that accompanies this paper (https://github.com/delshadv/BioFIND-data-paper/).

BIDS describes a way of organizing neuroimaging data by defining directory structures, a file naming scheme and file formats. The current version of BIDS does not specify how to handle multi-centre studies, so we simply combined data from both sites into the same directory and identified the site for each participant in the ‘participants.tsv’ file. The data passed the BIDS validator version 1.8.9 (https://bids-standard.github.io/bids-validator/).

According to BIDS, data are stored in their native format, and meta-data are stored in “sidecar” text files (.json,.txt,.tsv, etc.) to be both human- and computer-readable.

The top-level directory (Figure 1a) contains two separate BIDS directory: ‘MCIControls’ (approximately 319GB) and ‘TravelBrains’ (approximately 11GB; see below for information about the travelling brains). The ‘MCIControls’ directory includes 324 separate subdirectories (Figure 1b), one per participant, coded ‘sub-Sub’ followed by four digits for the unique participant number, matching the ‘participants.tsv’ file (see below). These directories contain the raw MEG+MRI data; the additional maxfiltered MEG data (see above) are stored in a mirrored format in the ‘derivatives’ sub-directory; furthermore, empty room data are placed within a directory called ‘sub-emptyroom’.

#### MCIControls BIDS directory

In addition to the above sub-directories, the MCIControls directory also contains the following files:

- The ‘participants.tsv’ file is a tab-separated text file that lists all the participants and associated information, as described in Table 2. Missing data are indicated by ‘n/a’.
- The ‘participants.json’ file is a tab-separated text file that describes all the column titles of ‘participants.tsv’.
- The ‘dataset_description.json’ is a JSON text file describing the dataset, including the name, license, authors and how to acknowledge.
- The ‘README’ file includes other important information about BioFIND data e.g., the CamCAN IDs of the control participants from Cambridge, to avoid duplication if a researcher wishes to analyse both BioFIND and CamCAN datasets.

#### Participant directories

As required by BIDS, within each participant’s ‘sub-Sub####’ directory (where # means one digit) is a sub-directory with a session name and number, in this case always ‘ses-meg1’ (since there is currently only one scanning session per participant). In this session directory are two further sub-directories, ‘anat’ and ‘meg’, which contain the anatomical MRI and MEG data respectively (Figure 1c).

The ‘anat’ folder contains the T1-weighted MRIs, stored in compressed (using GNU zip) NIfTI files, i.e, ‘*.nii.gz’ (where * represents some number of text characters). The international NIfTI format is read by many free software packages. The file name codes the participant number (‘sub-Sub####’), session (‘ses-meg1’) and data type (‘T1w’). Note that the faces on the MRIs images were removed using the FreeSurfer (Fischl, 2012) function (https://surfer.nmr.mgh.harvard.edu/fswiki/mri_deface). There is also a sidecar *.json file, created by hand, which is a text file containing useful meta-data about the T1 image, such as the anatomical MRI coordinate system, and in particular for MEG coregistration, the manually defined MRI indices for the Nasion, Left Peri-Auricular (LPA) and Right Peri-Auricular (RPA) fiducials.

The ‘meg’ folder contains the raw MEG data, in the native “FIFF” format developed by Neuromag (Elekta Instrumentation AB Stockholm). This format can be read by several free software packages. This file contains data from all the MEG channels, and additional EEG, EOG, ECG and several other miscellaneous channels (of no interest).

In addition, there are four accompanying sidecar files: *event.tsv, *channel.tsv, *coordsystem.json and *meg.json (Figure 1d). Although resting-state does not have any events, the *event.tsv is included to specify onset and duration of resting-state recordings within the file. The *channel.tsv file lists all the channels present in the data, while the *meg.json file encompasses other information about MEG acquisition parameters. The *coordsystem.json file contains required information about the coordinate system, unit as well as head coil coordinates. These sidecar files were created by the ‘data2bids’ function (http://www.fieldtriptoolbox.org/reference/data2bids/) of the FieldTrip software (Oostenveld et al., 2011).

#### Derivatives directory

The BIDS ‘derivatives’ sub-directory contains versions of the data that have been processed in some way. As noted above, we used MaxFilter to remove environmental noise from the raw data and recreate bad channels. We provide “maxfiltered” versions of the data because MaxFilter is proprietary software. The maxfiltered version of each subject’s MEG data is present in a FIFF file (sub-Sub####_ses-meg#_task-Rest_proc-sss_meg.fif) in the corresponding participant directory (Figure 1e). The files have the same name as the original raw MEG data, except for the addition element “proc-sss”, which indicates that the data have been processed with Signal-Space Separation (the method implemented in the MaxFilter software).

In addition to the *proc-sss_meg.json, *proc-sss_channel.tsv and *proc-sss_event.tsv files described above for the raw data, we also provide some additional text files that contain meta-data from the MaxFilter software. These are:

- sub-Sub####_ses-meg1_task-Rest_hpi.txt – the 3D locations (in MEG space) of the digitized headpoints
- sub-Sub####_ses-meg1_task-Rest_proc-sss_org.txt – coordinate of the centre of a sphere (in MEG space, relative to [0 0 0] as the origin of the helmet) fit to the above headpoints (after excluding points on the nose)
- sub-Sub####_ses-meg1_task-Rest_proc-sss_bad.txt – MEG channels determined as “bad” for each 10s segment of the data (and subsequently corrected by MaxFilter)
- sub-Sub####_ses-meg1_task-Rest_proc-sss_headpos.txt –the location of the centre of the head every 1s in quaternions, capturing head motion throughout the scan. The mean and standard deviation of head motion (relative to the initial location) have been extracted and put in the ‘participants.tsv’ file.
- sub-Sub####_ses-meg1_task-Rest_proc-sss_meg.log – the full log file output by MaxFilter, containing other information relevant to SSS.

The “meg_derivatives” sub-directory contains calibration (“sss_cal_*.dat”) and cross-talk (“ct_sparse_*.fif”) files that are needed by MaxFilter (see earlier). There is one each per site, although for CTB there are two calibration files, following a service on the scanner. The maxfilter_BIDS.m script in the GitHub repository encodes which calibration file (“sss_cal_CTB_1.dat” or “sss_cal_CTB_2.dat”) applies to which CTB participant.

The derivatives directory also contains an ‘anat’ sub-directory for every participant that contains another version of their T1-weighted MRI that has been “trimmed” rather than “defaced”. Unlike de-facing, this trimming keeps the nose, which is a useful feature for MEG-MRI coregistration when using digitised head-shapes (see (Bruña et al., 2022) for more information). The file name codes the participant number (‘sub-Sub####’), session (‘ses-meg1’), data type (‘T1w’) and the word (‘trimmed’).

#### Empty room directory

Empty room MEG files capture the environmental and system noise, and are located in a directory called ‘sub-emptyroom’ in the top level of MCIControls. This directory comprises different sessions for different years and acquisition sites, using the coding ‘ses-YYYYCBU/CTB’ (where YYYY means year of recording), followed by site name. In each session’s directory, there is a separate *scans.tsv sidecar file (sub-emptyroom_ses-YYYYCBU/CTB_scans.tsv) containing the date and time of the acquisition expressed in ISO8601 date-time format (YYYY-MM-DDThh:mm:ss); the additional ‘meg’ directory within each session includes corresponding *meg.fif, *channels.tsv and *meg.json files (Figure 1f).

#### Travelling Brains

Because of the potential importance of differences between scanners/recording sites, we also scanned 7 people on both scanners. These were young, healthy controls (not included in Table 1). Note that MRIs are only available for 4 of these travelling participants, and those MRIs come from different sites. Their MEG data are located in a separate BIDS directory named “TravelBrains”, which includes a “participants.tsv” file that includes sex, age, site, sImaging, group, move1CBU/CTB, move2CBU/CTB, Recording_timeCBU/CTB, Recording yearCBU/CTB. Note that in this case, the ‘site’ column refers to the laboratory in which MRI scan was acquired (since the MEG data were acquired in both sites). Because each participant has two FIFF files, one per site, there are now two session directories within each participant directory: ‘ses-megCBU’ and ‘ses-megCTB’. Each session directory holds ‘anat’ directory (when available) and ‘meg’ directory, like for the main BIDS repository described above.

### Example Analysis Pipeline

**Figure 2:**
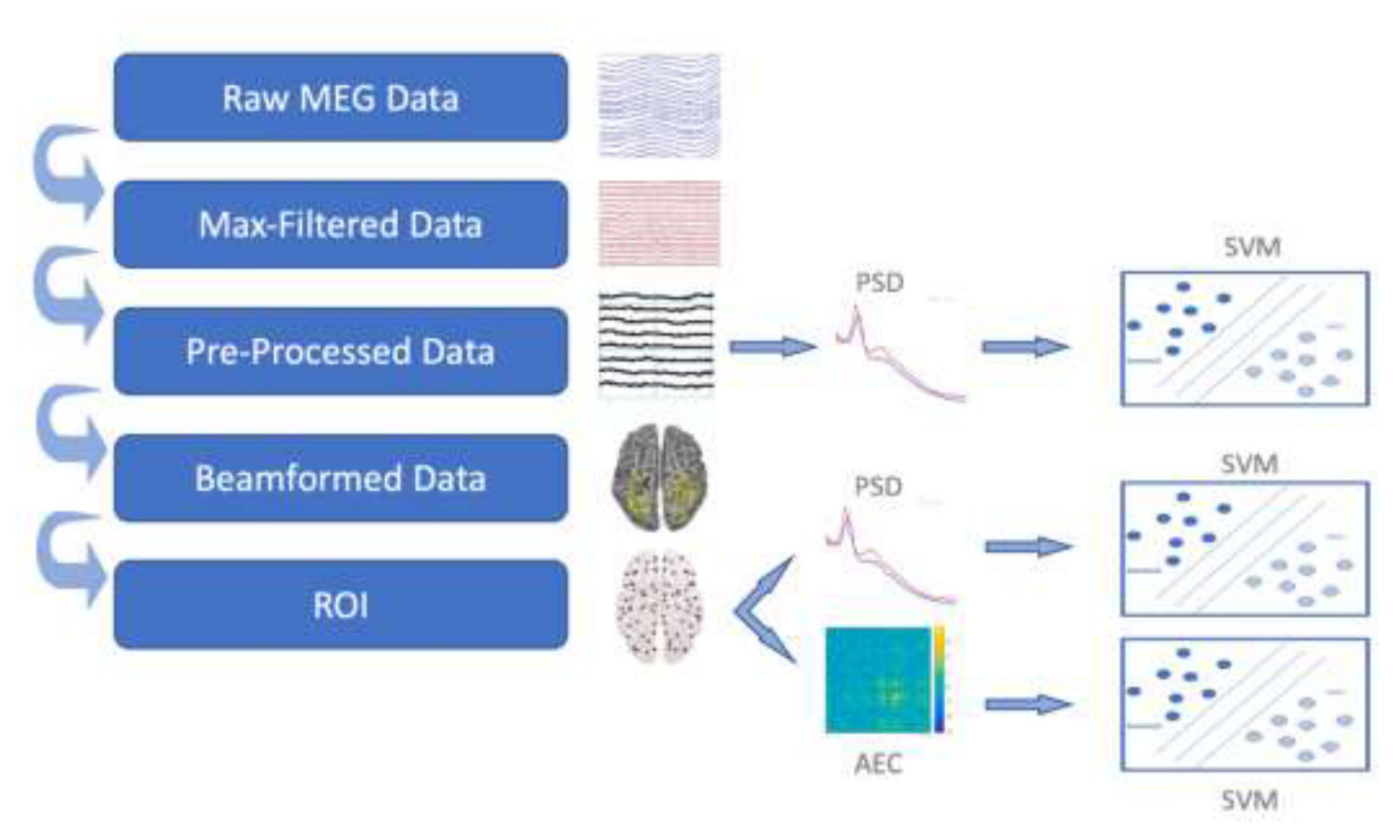
Paths from raw data to a classifier trained to distinguish MCI vs Control. Abbreviations: ROI, Region Of Interest; SVM, Support Vector Machine; AEC, Amplitude Envelope Correlation; PSD, Power Spectral Density

Figure 2 illustrates one possible analysis approach for using the MEG data to classify patients versus controls. The full code is available here on GitHub repository https://github.com/delshadv/BioFIND-data-paper/, specifically the MATLAB files: feature_extraction_test.m, preproc_beamform_ROI.m, repeated_CV.m and main.m.

#### Preprocessing

The maxfiltered data (in the derivatives directory) were read into MATLAB 2020b (MathWorks, Natick, MA, USA) using the SPM12 software (Litvak et al., 2011; Penny et al., 2006). The first 2 minutes were of data were then extracted, in order to equate the data duration across participants (if we had analysed all data available from every participant, this would have resulted in MEG features that reflected a longer duration of resting in some participants than in others, introducing a further source of between-participant variance, e.g., in their level of sleepiness). It is worth mentioning that the minimal length of data required to yield a robust feature in M/EEG studies is still unclear. A recent study showed intrinsic brain activity can be robustly estimated when derived from relatively short segments of 30s to 120s of resting-state data, regardless of equipment technology and resting-state paradigm (Wiesman et al., 2021). Nonetheless, future analyses might want to maximize the data available from everyone, and conduct extra analyses that control for potential differences in sleepiness, or increase the minimum slightly, depending on what duration of data is optimal.

Data from the 102 magnetometers and 204 gradiometers were then down-sampled to 500 Hz and band-passed filtered from 0.5-150 Hz via a high-pass filter followed by a low-pass filter. This frequency range is often assumed to reflect the range of oscillatory electrical activity used by the brain and detectable by extracranial techniques like MEG. The filter type was a Butterworth IIR filter with the order of 5, as implemented in SPM’s spm_eeg_filter.m function. The continuous data were then epoched into 2s windows, and atypical epochs were automatically marked as “bad” using the artefact detection function in the OSL toolbox (https://ohba-analysis.github.io/osl-docs/). This uses a Generalized ESD test to identify “outlier” epochs, which are likely to contain noise aritfacts like muscle activity. While more sophisticated techniques like ICA could be used to further identify artifactual noise sources, manual inspection of such potential noise components becomes laborious with the large numbers of participants as here, and, as noted in the Methods, EOG and/or ECG data were not available on all participants to help automate identification of ocular and/or cardiac artifacts. Note also that eyes were closed, which will abolish blinks and minimize saccades (though residual eye movements are likely to remain; (Mannan et al., 2018; Shimazono et al., 1965), while the dominant power in ECG is typically around 1Hz, which is below the lowest frequency analyzed here.

As shown in Table 1, there was trend for the number of bad epochs to be slightly greater in patients than controls (note that, since the number of bad epochs is a property of the preprocessing approach, rather than a property of the raw or maxfiltered data, that number is not included in the participants.tsv file). While data from bad epochs were excluded, the number of bad epochs could also be used as a further confounding covariate to adjust for overall data quality.

The identification of bad epochs is influenced by the epoch length, which needs to be long enough to capture at least one cycle of the lowest frequency of interest. The number of cycles also affects the accuracy of power estimation (Cohen, 2014). The lowest frequency analysed here was 8 Hz, meaning that our 2s epochs allowed 16 cycles.

The remaining “good” epochs were used to calculate power spectral density (PSD) for each channel every 0.5Hz (using MATLAB’s ‘periodogram’ function), which was then averaged over epochs. Given that the absolute power depends on the position of the cortical sources relative to the sensors (i.e., head position), we normalised power by dividing each channel’s PSD by its mean power across frequencies and channels. Given the different scaling of the magnetometers and gradiometers, this normalisation was done separately for each sensor-type. For each channel, we then averaged the normalised power over frequencies in the range 8-12Hz and 13-30Hz, reflecting the distribution over the scalp of power of the “Alpha” and “Beta” rhythms respectively, given that previous studies have shown one or both to differ in dementia patients (Baker et al., 2008; Jelic et al., 2000; López-Sanz et al., 2016; Michels et al., 2017).

The distribution of normalised Alpha and Beta power over the 102 magnetometers and 204 gradiometers was then used to classify patients versus controls (see below). For comparison, we repeated this classification using power estimates for the cortical sources, estimated from the sensor data and the MRI, as explained in the next section.

#### Source reconstruction

Given that the MEG signal depends on the position and geometry of the head, a more accurate method to estimate brain activity is to construct a “forward” model (Bertero et al., 2021; Brette and Destexhe, 2012; Henson et al., 2009; Mosher et al., 1999). This model specifies how the magnetic fields produced by electrical dipoles in the cortex appear at the sensors. It requires the location and orientation of each dipole, the shape of the cortex and skull (extracted from an MRI) and information about the position of each sensor relative to the head. This model can then be inverted to estimate the cortical currents, subject to additional constraints for this “ill-posed” inverse problem.

The first step in this source reconstruction is to coregister the MRI to the MEG data, which we did by minimising the error between the digitised head-points and the scalp surface extracted from the MRI. We did this using SPM12, after excluding points on the nose, since the nose is not always captured in the MRI (the residual error in the 3 anatomically defined fiducials can be used as an independent measure of coregistration accuracy). Note that this meant excluding the 15 participants for whom no MRI is available.

Once coregistered, we modelled 3559 cortical sources every 8mm within the brain, and estimated how a dipolar source at each location, oriented normal to the cortical surface, would appear to each sensor, using a single-shell “boundary element” model. We then estimated those sources using a scalar, Linear-Constrained Minimum Variance (LCMV) beamformer implemented in the OSL toolbox. This was estimated from the sensor time-series (from all “good” epochs) across a broadband of frequencies from 0.5 to 48 Hz. Each source was then assigned to one of 38 regions of interest (ROIs) based on (Colclough et al., 2016, 2015), and principal component analysis was used to extract a single representative time-series per ROI. Before LCMV beamforming, two sensor types i.e., gradiometers and magnetometers were normalised to have equal variances over time so that they can contribute equally to the beamformer calculation. This is done using “osl_normalise_sensor_data.m” function from the OSL toolbox. Similar to sensor features, Alpha and Beta powers were then calculated at ROI level, to produce 38 features per participant.

For the final type of feature, we also estimated functional connectivity between the 38 ROIs, using a standard approach in the OSL software. Briefly, each ROI’s time-series was first orthogonalized with respect to all others using symmetric orthogonalization (Colclough et al., 2015), to reduce the influence of residual leakage between sources. A Hilbert transform from 8-12 Hz was then applied to the ROI time-series, and the amplitude envelope was calculated. This was then downsampled to 1 Hz, and the Pearson correlation coefficient was estimated between all pairs of ROIs, producing (38×37)/2=703 unique estimates of functional connections.

#### Confounds and Classification

The data features were adjusted for various potential confounds in Table 2 by regressing out linear effects of each covariate (after missing values of these covariates were first imputed using their mean value).

The sensor or source features derived above were used to train and test a Support Vector Machine (SVM) (Cortes and Vapnik, 1995) using cross-validation with 10-folds, and performance averaged over 100 permutations. Since the number of patients and controls differed, performance was assessed by “balanced accuracy”, i.e, the average of the percentage of patients classified correctly and the percentage of controls classified correctly.

### Results

In the first analysis, we examined MCI-Control classification performance when using all sensors, i.e., 712 features per participant, consisting of normalised Alpha and Beta power across the 306 channels. More specifically, we compared classification as a function of which covariates from Table 2 were removed from the data before classification. Baseline performance was 67.8% when using no covariates (Table 3).

The potential covariates from Table 2 can be divided into at least two types. The first type are variables that may differ between patients and controls, and may affect the MEG features, but would not be an obvious or direct consequence of AD. These variables should be covaried out, so that classification is not based on an independent, accidental property. These included recording site, time of day of recording, duration of any previous tasks, number of bad epochs and head motion (mean and standard deviation of translation).^1^ When adjusting the data for these types of potential confound, performance dropped to 66.9% (Table 3), but was still clearly above chance (50%).

The second type of covariate are those that could affect MEG features, but could also be causally related to AD. Here, these were age, sex and education. The rationale for covarying out these covariates is less clear. To take the example of education: the level of education could affect whether someone develops AD later in life (e.g, owing to other lifestyle choices). Therefore, when adjusting the MEG data for education, one could remove effects in the MEG data that are truly related to AD.^2^ It is therefore informative to consider classification results both with and without adjusting for this second type of covariate. When adjusting for this type of covariate, in addition to the first type above, performance dropped to 59.4%. For reasons stated above, this drop in classification does not necessarily mean that MEG features are only very weakly related to AD, and ideally independent justification would be needed before adjusting for variables like these.

In a second set of analyses, we compared classification performance based on different types of MEG features: sensor power relative to source power, and source power relative to source connectivity (see Methods). For these analyses, we only used the 309 participants who also had an MRI (to enable more accurate source localisation), and we adjusted for the first type of covariates listed in the previous set of analyses. As shown in Table 4, classification based on source power or source connectivity was worse than that based on sensor power. While this suggests that estimating cortical sources does not improve classification in this case, it is important to note that this case is specific to the 38 ROIs used here, and the specific measure of connectivity. It is also important to note that the total number of MEG features varied considerably across analyses. It is possible that different approaches to source reconstruction (e.g. greater number of ROIs) and different measures of connectivity (e.g. phase-locking value,(Pusil et al., 2019) would produce higher classification performance (as might the use of other types of classifier). Again, our purpose is not to claim this is the best classification possible, nor that we have chosen the best method possible, but to illustrate the type of analyses one could do on this dataset, and provide some benchmarks for future analyses.

### Discussion

The main purpose of the present paper is to describe the largest currently-available MEG resting-state data for the study of early dementia. This will hopefully enable scientific and clinical inferences beyond those possible from previous MEG studies, which have tended to focus on relatively small samples (Yang et al., 2019), and/or been unable to share their data in order to compare analyses. We report results of one pipeline for preprocessing the MEG data and classifying participants on the basis of their MCI status, but emphasize that we do not claim this is the best pipeline; our purpose was just to show that classification (60-68%, depending on covariates) was at least above chance (50%), comparable with previous studies using resting-state MEEG data and similar classifiers (Dimitriadis et al., 2018; Hughes et al., 2019; Pineda-Pardo et al., 2014; Yang et al., 2019), and to provide benchmarks for future analyses on these data. While we hope that future analyses will enable higher classification levels, we do not expect classification to ever reach 100%, because there are sure to be errors in some of the class labels, since these are based on a clinician’s diagnosis, which is imperfect. For example, not all of the MCI group are likely to have AD pathology (Petersen et al., 1999), while a proportion of older controls may have latent AD pathology without symptoms.

Future alternative approaches include, for example, using other types of MEG features (e.g., different frequency bands, different measures of connectivity), other types of machine learning (e.g., random forest, ensemble classifiers, deep learning) and improved methods for pre-processing (de-noising) the MEG data (e.g., using ICA to remove cardiac and other artefacts), or for reconstructing the cortical sources (e.g., improved forward models or inverse methods). Future studies could also distinguish between the subset of MCI patients who subsequently developed probable AD (converters), or further explore the role of potential confounds like education. Furthermore, there are aspects of the dataset that we did not use, such as the empty-room data (to better estimate MEG noise) and the travelling brains data (to better explore potential site differences); and while we used the T1-weighted structural MRI to assist source localisation, the MRI scans could also be used for classification, to compare accuracy with that achieved by MEG, or combined with the MEG in multi-modal classification.

#### Caveats

There are several limitations of the current dataset. First is the fact that the data come from two different sites. While this can be seen as an attraction, in terms of testing the generalisation across sites that would be necessary if MEG were to be used across the world to aid dementia classification, there are multiple types of site differences. Although the MEG machine model was identical, individual scanners differ in their noise levels (e.g, sensor tuning) and their magnetic environment (e.g, in terms of MSR effectiveness). Ideally, we would be able to provide more empty-room recordings that were closer in time to each participant’s recording (as recommended by (Gross et al., 2013; Pernet et al., 2020), but this was not possible. Furthermore, though following similar international guidelines, the precise definition of “MCI” is likely to differ across clinicians in the two countries, as did the recruitment method and therefore likely nature of controls (see “Participants” section above for full details). Though the resting-state instructions were very similar, the state of the participants varied, since some had performed tens of minutes of cognitive tasks prior to resting. While we provide this number of minutes, it is possible that different tasks had different levels of fatigue, etc, which will contribute to noise in the data. The groups also differ in other respects, such as age and education. While we provide information on, and described potential adjustment for, as many of these potential confounds as we can, it is possible that there are other confounding covariates on which we do not have data. Though more homogeneous data could be achieved from matched sampling in individual studies, the likely heterogeneity in real-world clinics makes it important to also study the potential effects of these confounding variables. Other more specific limitations are that 15 participants are missing MRI data; 41 patients are missing information about subsequent AD progression; 2 participants only had 2 minutes of data.

#### Future

While this is the first public release of the BioFIND dataset, we intend to continue to grow the repository in future. We already know of several projects at both sites that are collecting new MEG data on MCI and controls, and would welcome MEG data from other sites too. Given the growing realisation of the importance of “big data” for goals like dementia detection, future additions to the BioFIND dataset may come from larger, more homogeneous projects, and it may be helpful to add further sub-groupings (eg “project” in addition to site), to help classifiers to generalise across projects. We would also like to follow-up the current participants to update those (including controls) who later converted to a dementia diagnosis. Furthermore, we could add data from participants with later stages of dementia, e.g. a more confident (clinical) diagnosis of AD, as supported by other biomarkers. Though our aim is to evaluate the ability of MEG to detect early stages of the disease, having late-stage cases is likely to help train classifiers (assuming they have the same MEG signatures; though see (Pusil et al., 2019), for possible nonlinear changes as the disease progresses).

## Figures and Tables

**Table 1:** Summary of data characteristics.

Data Characteristic	Groups		T/χ^2^-test	
	Controls	MCI	T/χ^2^ and p value
**Site (CBU/CTB)**	91/75	68/90	χ^2^ = 4.04 p = 0.04
**Sex (M/F)**	82/84	80/78	χ^2^ = 0.01 p = 0.91
**Age (years)**	71.3 (7.0)	72.9 (6.7)	T = -2.07 p = 0.04
**Education (years)**	14.5 (4.4)	10.8 (5.3)	T = 6.70 p <.001
**MMSE (/30)**	28.8 (1.2)	26.1 (2.8)	T = 11.11 p <.001
**Recording Duration (seconds)**	481.5 (262)	180.0 (305)	Z = 4.19 p <.001
**Recording Hour (24h)**	12.8 (2.4)	12.6 (2.1)	T = 1.13 p = 0.26
**Recording Year (calendar)**	2013.8 (2.4)	2012.4 (2.5)	T = 5.37 p <.001
**Previous Tasks (minutes)**	0 (23.3)	0 (40.0)	Z = -1.15 p = 0.25
**Mean of head translation (mm)**	1.9 (1.8)	2.3 (2.1)	T = -1.59 p = 0.11
**SD of head translation (mm)**	1.1 (1.1)	1.2 (1.1)	T = -1.03 p = 0.30
**Number of bad epochs**	4.1 (2.8)	4.7 (3.8)	T = -1.74 p = 0.08

Numbers shown for Site and Sex; medians (with interquartile range in parentheses) shown for Recording Duration and Previous tasks; means (with standard deviation in parentheses) shown for all others. Values for each individual participant are given in the “participants.tsv” file. Abbreviations: CBU, Cognition & Brain sciences Unit; CTB, Centre for Biomedical Technology; MMSE, Mini-Mental State Examination; MCI, Mild Cognitive Impairment; M, Male; F, Female; SD standard deviation.

**Table 2:** Fields in participants.tsv file

Column title	Description
participant_id	Name of participant sub-directory, e.g. “sub-Sub0001”
group	Either ‘control’ or ‘patient’ (MCI)
site	Either ‘CTB’ (Madrid site) or ‘CBU’ (Cambridge site)
sex	Either ‘F’ (Female) or ‘M’ (Male)
age	Integer years (ranging from 52 to 95)
MMSE	Mini-Mental State Examination
sImaging	‘MRI’ if MRI available (‘n/a’ if not). Note that 15 participants did not have MRIs available.
Converters	Only applies to patients: those who later progressed to AD: ‘1’ = converted, ‘0’ = not converted, ‘n/a’ = data unavailable
Recording_year	MEG Recording year
Recording_time	MEG data acquisition time of day (hour)
Edu_years	Total years in education (primary, secondary, and tertiary)
Move1	Mean of head translation during MEG scan (from MaxFilter), relative to initial position
Move2	Standard deviation of head translation during MEG scan (from MaxFilter)
Pre_Task	Number of minutes of (any) tasks before resting-state recording started

**Table 3:** Mean of 10-fold cross-validation performance across 100 permutations (with standard deviation in parentheses), using normalised power across all sensors in Alpha and Beta range for all N=324 participants, as a function of covariates used (see text for definition of type 1 and type 2 covariates).

Type of Covariate	No. of Features	Accuracy (%)
No covariates	712	67.8 (1.4)
Type 1 covariates	712	66.9 (1.4)
Type 1+2 covariates	712	59.4 (1.4)

**Table 4:** Mean of 10-fold cross-validation performance across 100 permutations (with standard deviation in parentheses), using normalised power across all sensors in Alpha and Beta range for all N=309 participants with an MRI, as a function of MEG feature

MEG Feature	No. of Features	Accuracy (%)
Sensor power	712	66.1 (1.3)
Source power	76	65.2 (1.4)
Source connectivity	1406	64.8 (1.3)

## Data Availability

The custom-written code to implement all validation analyses is available on GitHub (https://github.com/delshadv/BioFIND-data-paper). If one analyses data through DPUK there is no need to clone the GitHub repository since all codes and data have been already uploaded there and are ready to use. Some imaging analysis toolboxes, written in MATLAB, are necessary to reproduce the result of this paper are also available on DPUK e.g., OSL, SPM, Fieldtrip. Any other software and/or toolboxes can be provided by DPUK if requested. Please note that OSL is a Linux-based toolbox thus, to obtain necessary information on how to use Linux through DPUK please read “instructions.md” in the GitHub repository or ask DPUK. Note that there is no internet connection on DPUK, so any file should be accessed by an in/out file request on https://portal.dpuk.ukserp.ac.uk in case one needs to have any other resources, such as other GitHub repositories, libraries, etc. The starting point to run validation analysis is /biofind/data/Code/BioFIND-data-paper/preproc_beamform_ROI.m script. However, there is a MATLAB script named “main.m”, including all steps in one place. For those who wish to use our preprocessing and feature extraction steps, e.g., who are only interested in the machine learning aspect, all MEG features are available in comma-separated value (.csv) files in the “derived” directory within the GitHub repository. These are: “Sensor_RP.csv” (relative power in sensor space), “Source_RP.csv” (relative power in source space) and “AEC.csv” (amplitude envelope correlation).

## Highlight

- Largest freely-available resting-state MEG data to study dementia
- Contains MEG and T1-weighted MRI data from two sites in BIDS format

## Footnotes

1 We appreciate that some of these could still have conceivable relationships with AD - for example, AD could conceivably affect head motion, or the number of bad epochs. Note also that, because we only considered the first 2 minutes of data, the total duration of recording for each participant in Table 1 could not affect the MEG features, so was not used to adjust the data.

2 MMSE is arguably an extreme case of this type of covariate, since not only are MMSE scores largely a consequence of AD, but they are also part of the information used by clinicians to define the MCI label in the first place. Therefore adjusting for MMSE is likely to remove the majority of true differences in brain activity that are related to AD (i.e, MMSE and MEG features are correlated consequences of the same cause).
